# An Acetone Microsensor with a Ring Oscillator Circuit Fabricated Using the Commercial 0.18 μm CMOS Process

**DOI:** 10.3390/s140712735

**Published:** 2014-07-17

**Authors:** Ming-Zhi Yang, Ching-Liang Dai, Po-Jen Shih

**Affiliations:** 1 Department of Mechanical Engineering, National Chung Hsing University, Taichung 402, Taiwan; E-Mail: d099061005@mail.nchu.edu.tw; 2 Department of Civil and Environmental Engineering, National University of Kaohsiung, Kaohsiung 811, Taiwan 320, Taiwan; E-Mail: pjshih@nuk.edu.tw

**Keywords:** acetone microsensor, α-Fe_2_O_3_, ring oscillator circuit

## Abstract

This study investigates the fabrication and characterization of an acetone microsensor with a ring oscillator circuit using the commercial 0.18 μm complementary metal oxide semiconductor (CMOS) process. The acetone microsensor contains a sensitive material, interdigitated electrodes and a polysilicon heater. The sensitive material is α-Fe_2_O_3_ synthesized by the hydrothermal method. The sensor requires a post-process to remove the sacrificial oxide layer between the interdigitated electrodes and to coat the α-Fe_2_O_3_ on the electrodes. When the sensitive material adsorbs acetone vapor, the sensor produces a change in capacitance. The ring oscillator circuit converts the capacitance of the sensor into the oscillation frequency output. The experimental results show that the output frequency of the acetone sensor changes from 128 to 100 MHz as the acetone concentration increases 1 to 70 ppm.

## 1. Introduction

Gas sensors are important devices for several industrial, environmental, foodstuff and biomedical applications, *etc*. Most of the methods for detecting diabetes are invasive ones that take blood samples and inspect blood sugar, but their deficiencies are painful for patients with diabetes. The breath of diabetes patients had a higher acetone concentration than that of healthy persons. Therefore, acetone sensors could be used to detect diabetes [1]. The benefits of this method are its non-invasiveness and non-painful inspection for diabetic patients.

The advantages of gas microsensors include small volume, low cost, high performance and easy mass-production [2]. Recently, micro-electromechanical system (MEMS) technology has been used to develop various gas microsensors. For instance, Kim *et al.* [3] fabricated a hydrogen gas microsensor using MEMS technology. The sensor contained a sensitive material, a Pt heater and Pt electrodes on an alumina substrate. The sensitive material was a SnO_2_-Ag_2_O-PtO_x_ composite prepared by the sol-gel method. Pandya *et al.* [4] proposed an ethanol microsensor manufactured by MEMS technology. The sensor was composed of a sensitive film, aluminum electrodes and a nickel heater on a silicon substrate. The ethanol sensitive film was zinc oxide nanoflakes, and the film was deposited on the aluminum electrodes using thermal evaporation. Zhang *et al.* [5] utilized MEMS technology to make a gas sensor with heaters for detecting volatile organic compounds. The sensitive film of the sensor was zinc oxide nanosheets. The sensor had a high sensitivity to acetaldehyde and formaldehyde at working temperature of 220 °C.

Several studies have employed MEMS technology to fabricate acetone microsensors. For instance, Zeng *et al.* [6] proposed an acetone microsensor manufactured using MEMS technology. The sensitive material of the sensor was ZnO nanorods prepared by an aqueous solution method. The sensor was a resistive type, and it had a response of 30 at 100 ppm acetone. Liu *et al.* [7] reported a gas microsensor for detecting acetone, and the sensor was fabricated using micromachining technology. The sensitive film of the sensor was SnO_2_-TiO_2_ doped with Ag prepared by the sol-gel method, and the film was coated onto the interdigitated electrodes on an alumina substrate using screen printing. Srivastava *et al.* [8] utilized MEMS technology to develop a gas sensor for sensing acetone, methanol and propanol. The sensor consisted of silver electrodes, a sensing layer and a heater on an alumina substrate. The sensing layer was SnO_2_ doped with PbO synthesized by sol-gel method and coated on the silver electrodes. Inyawilert *et al.* [9] manufactured a gas microsensor for detection of acetone using micromachining technology. The sensor contained a sensitive film and Au electrodes on Al_2_O_3_ substrate, which the sensitive film was In_2_O_3_ fabricated by a sparking method.

The smallest sensing concentration of the acetone sensor proposed by Liu *et al.* [7] was 200 ppm, and the sensor was not integrated with circuitry on chip. The acetone sensor without circuits on chip, developed by Srivastava *et al.* [8], had a smallest sensing concentration of 500 ppm. Inyawilert *et al.* [9] presented an acetone sensor also without circuits on chip, and its smallest sensing concentration was 50 ppm. Acetone sensors for medical application, such as detecting diabetes, require having a capability of low concentration detection under 1 ppm, so the sensors described in [7–9] are not suitable for application in detecting diabetes patients. In this work, we develop an acetone sensor with a smallest sensing concentration of 1 ppm. The acetone microsensors [6–9] were not integrated with circuitry on-a-chip. The advantages of microsensors with circuitry on-a-chip are low noise, high performance and low package cost [2]. In this study, an acetone sensor integrated with a ring oscillator circuit on-a-chip is developed.

Various microsensors and microactuators have been fabricated using the commercial CMOS process [10–14]. Microdevices manufactured by this process can be integrated with circuitry on-a-chip [15–17]. Hu *et al.* [18] used the commercial 0.18 μm CMOS process to manufacture a humidity microsensor with a readout circuit on-a-chip, and the sensor was a resistive type. The sensitive material of the humidity sensor was titanium dioxide, and the readout circuit converted the resistance of the sensor into the output voltage. In this work, we employ the same process to develop a capacitive acetone microsensor with a ring oscillator circuit on-a-chip. The sensitive material of the acetone sensor is α-Fe_2_O_3_, because it had good sensitivity to acetone vapor [19,20]. The ring oscillator circuit converts the capacitance into the output frequency, which the output signal has a potential for application in wireless communication system. The acetone sensor has a capability to detect a low concentration of 1 ppm acetone. The sensor needs a post-process [21–23] to deposit the sensitive material. The post-process consists of removing the sacrificial oxide layer and coating the α-Fe_2_O_3_ film.

## 2. Structure of the Acetone Sensor

**Figure 1.**
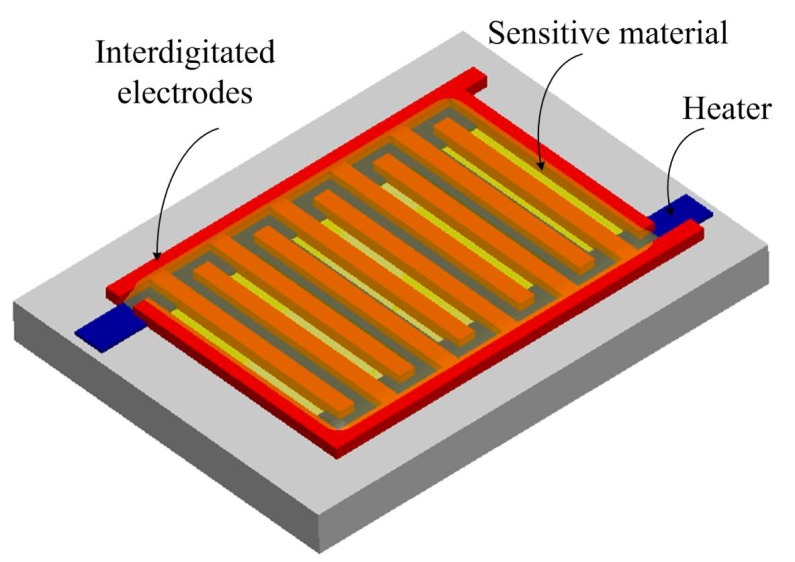
Schematic structure of the acetone microsensor.

Figure 1 shows schematic structure of the acetone microsensor. The structure of the sensor contains a sensitive material, a polysilicon heater and interdigitated electrodes. The length, width and thickness of the interdigitated electrodes are 400 μm, 20 μm and 6 μm, respectively, and the gap between the electrodes is 10 μm. The polysilicon heater is located under the interdigitated electrodes, and it is employed to provide a working temperature of 265 °C to the sensitive material. The sensitive material of the sensor is α-Fe_2_O_3_ coated on the interdigitated electrodes. The sensor is a capacitive type. The capacitance of the sensor produces a variation when the sensitive material of α-Fe_2_O_3_ adsorbs or desorbs acetone vapor. The α-Fe_2_O_3_ is a n-type semiconductor. The sensing mechanism of α-Fe_2_O_3_ to acetone had been reported [19]. The reaction is based on the α-Fe_2_O_3_ surface adsorbed oxygen molecules, and the conduction band of the α-Fe_2_O_3_ is ionized by oxygen ions (O^−^, O^2−^ or O_2_^−^). The release electrons are trapped in the conduction band of the α-Fe_2_O_3_, resulting in the dielectric of the α-Fe_2_O_3_ changes.

**Figure 2.**
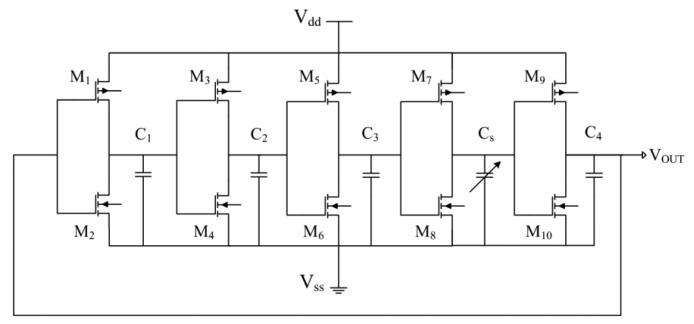
Ring oscillator circuit for the acetone microsensor.

**Figure 3.**
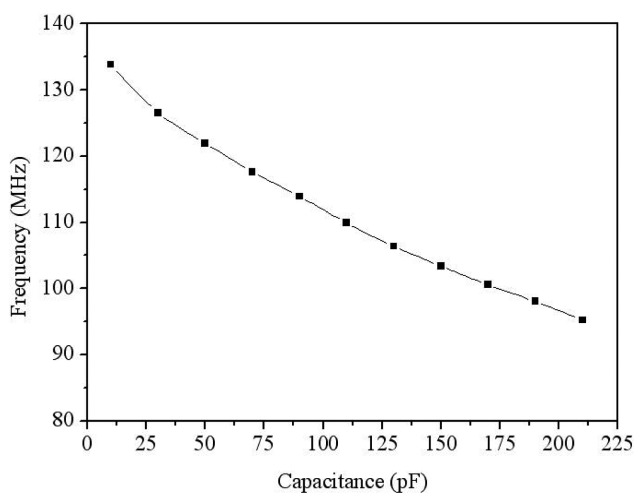
Simulation results of the output frequency for the circuit.

A ring oscillator circuit is utilized to convert the capacitance variation of the sensor into an oscillation frequency. Figure 2 illustrates the ring oscillator circuit for the acetone microsensor. The circuit is five-stage ring oscillator [24] where *V_dd_* is the input voltage; *V_ss_* is the ground; *V_out_* is the output voltage of the circuit; *C_1_*, *C_2_*, *C_3_* and *C_4_* are the load capacitance; *C_s_* is the capacitance of the acetone sensor; *M_1_*, *M_3_*, *M_5_*, *M_7_* and *M_9_* are p-channel metal oxide semiconductor (PMOS); *M_2_*, *M_4_*, *M_6_*, *M_8_* and *M_10_* are n-channel metal oxide semiconductor (NMOS). The simulation software, HSPICE (Synopsys lnc., Mountain View, CA, USA), is employed to simulate the performances of the circuit. Figure 3 illustrates the simulation results of the circuit for the acetone sensor. In the simulation, the input voltage *V_dd_* of 3 V was adopted, and the capacitance of the sensor changed from 10 to 210 pF. The temperature was set at 265 °C. The simulation results showed that the output frequency of the circuit varied from 143 to 98 MHz when the capacitance of the sensor changed from 10 to 210 pF.

## 3. Preparation of the Sensitive Material

The acetone sensitive material was fabricated by a hydrothermal method. The preparation steps of α-Fe_2_O_3_ were as follows [19,20]: ferric chloride (FeCl_3_·6H_2_O, 4 g) and ammonium sulfate ((NH_4_)_2_SO_4_, 0.1 g) were dissolved in deionized water (50 mL) with vigorous stirring for 2 h until the solution was mixed uniformly. The concentration of α-Fe_2_O_3_ in the solution was 25 wt%. The mixing solution was transferred into a Teflon-lined stainless-steel autoclave, where the solution was sealed and maintained at 120 °C for 12 h. After completion of the reaction, the result was filtered and washed with deionized water and ethanol. Then, the product was coated on the substrate, followed by calcination at 400 °C for 2 h.

**Figure 4.**
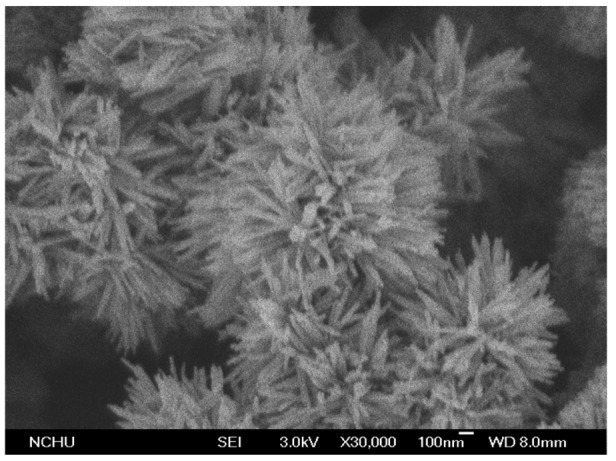
SEM image of the α-Fe_2_O_3_.

**Figure 5.**
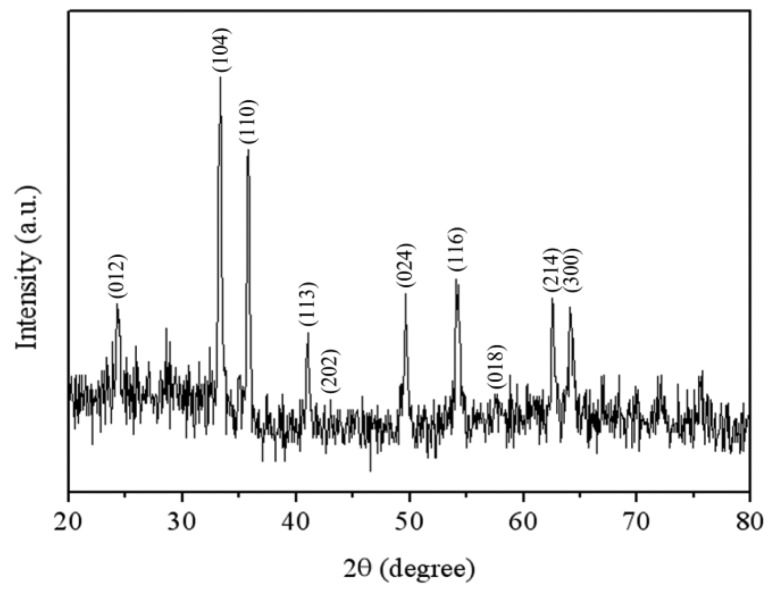
XRD pattern of the α-Fe_2_O_3_.

**Figure 6.**
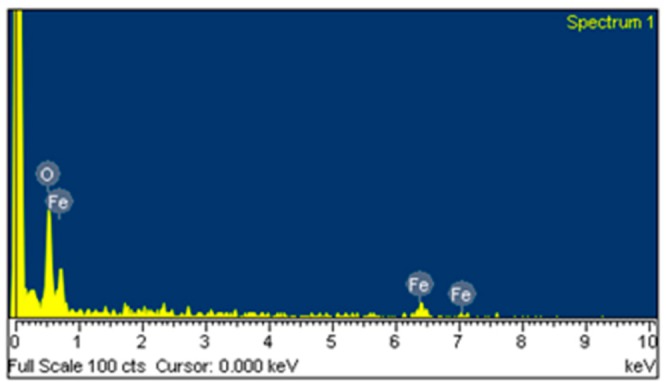
Elements of the α-Fe_2_O_3_ measured by energy dispersive spectrometry.

Scanning electron microscopy (SEM, JEOL JSM-6700F, Tokyo, Japan) was used to measure the surface morphology of the α-Fe_2_O_3_. Figure 4 shows a SEM image of the α-Fe_2_O_3_. The α-Fe_2_O_3_ exhibits urchin-like nanorod shapes. Figure 5 shows the X-ray diffraction (XRD; pattern of the α-Fe_2_O_3_. The diffraction peaks showed that the material was α-Fe_2_O_3_ structure. The elements of the α-Fe_2_O_3_ were measured by an energy dispersive spectrometer. Figure 6 shows the measurement results of the α-Fe_2_O_3_ using an energy dispersive spectrometer. The results depicted that the α-Fe_2_O_3_ was composed of 32.09 wt% O and 67.91 wt% iron.

## 4. Fabrication of the Acetone Sensor

**Figure 7.**
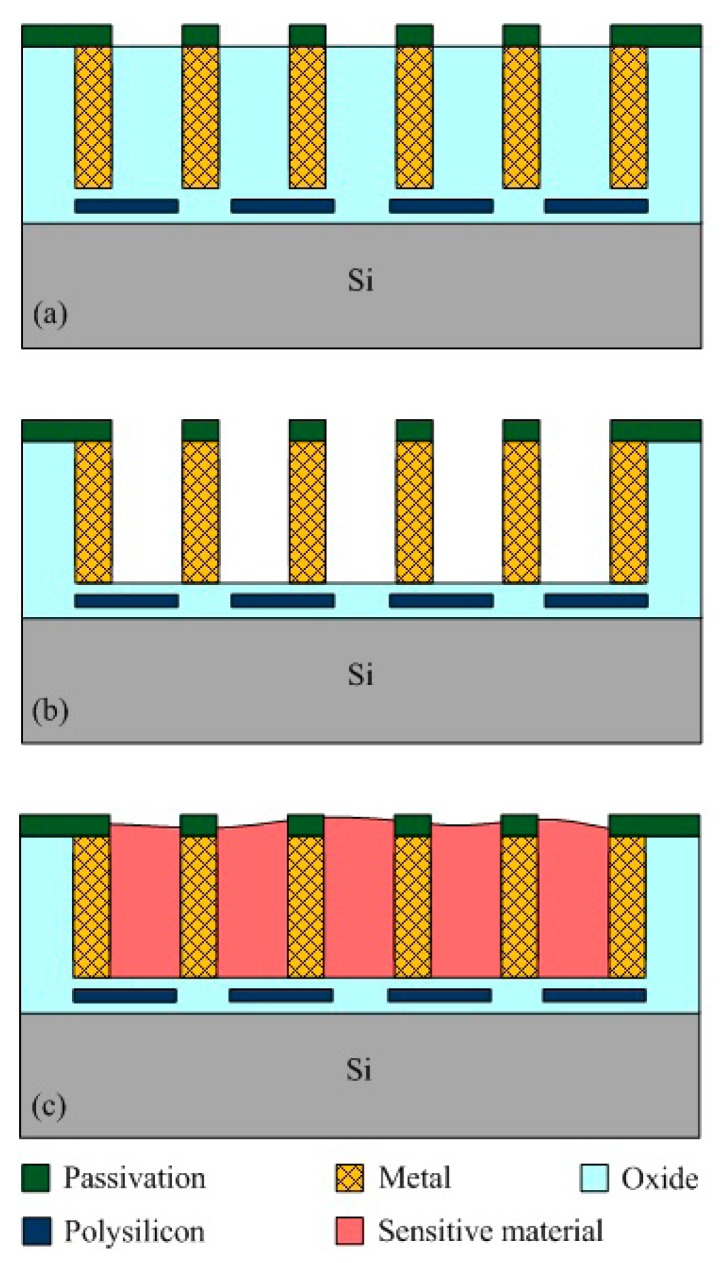
Fabrication flow of the acetone microsensor, (**a**) after the CMOS process; (**b**) removing the sacrificial oxide layer; (**c**) coating the sensitive α-Fe_2_O_3_ material.

**Figure 8.**
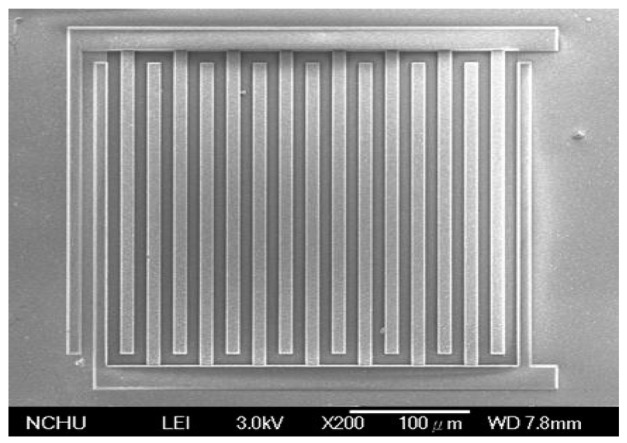
SEM image of the acetone sensor after the wet etching.

**Figure 9.**
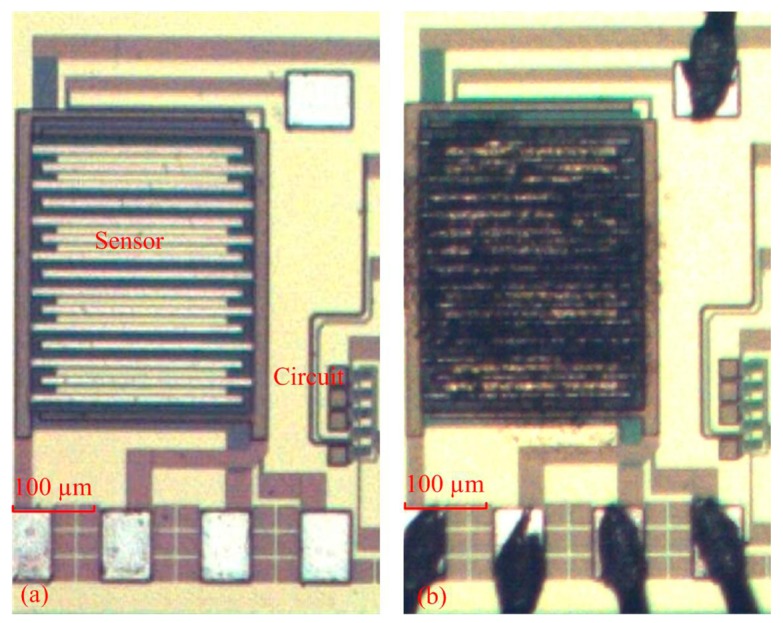
Optical image of the acetone microsensor, (**a**) before the post-process; (**b**) after the post-process.

The acetone microsensor was fabricated using the commercial 0.18 μm CMOS process of Taiwan Semiconductor Manufacturing Company (TSMC, Taipei, Taiwan). Figure 7 illustrates the fabrication flow of the acetone microsensor. Figure 7a presents the cross-sectional view of the acetone sensor after the CMOS process. The thickness of passivation, metal and polysilicon was 0.75 μm, 6.43 μm and 0.2 μm, respectively. The acetone sensor needed a post-processing step to etch the sacrificial layer, and to coat the sensitive material of α-Fe_2_O_3_ after completion of the CMOS process. The sacrificial layer between the interdigitated electrodes was silicon dioxide. Figure 7b displays that the sacrificial layer of silicon dioxide is removed. A wet etching of buffer etch oxide (BOE) etchant was used to etch the sacrificial oxide layer [25–27] and to expose the interdigitated electrodes. Figure 8 shows a SEM image of the acetone sensor after the wet etching. The interdigitated electrodes were exposed. Figure 7c shows that the sensitive material of α-Fe_2_O_3_ is coated. The α-Fe_2_O_3_ was dropped onto the interdigitated electrodes using a precision-control micro-dropper. The volume of the drop was about 1.5 × 10^−3^ mL. The thickness of the coating was about 10 μm. Finally, the α-Fe_2_O_3_ was sintered at 400 °C in air for 2 h. Figure 9a shows an optical image of the acetone sensor before the post-process. Figure 9b an optical image of the acetone sensor after the post-process.

## 5. Results and Discussion

A spectrum analyzer, a test chamber, an infrared thermometer and a LCR meter were used to measure the performance of the acetone microsensor. The infrared thermometer was utilized to detect the heater temperature. The LCR meter was adopted to measure the capacitance of the acetone sensor, and the spectrum analyzer was used to record the output frequency of the oscillator circuit. The test chamber contained a calibration acetone sensor, a control valve and a pump. The acetone concentration in the test chamber could be tuned by the control valve. The pump is used to exhaust the acetone in the test chamber after completion of testing. The calibration acetone sensor monitored the acetone concentration in the test chamber. The acetone microsensor was set in the test chamber.

**Figure 10.**
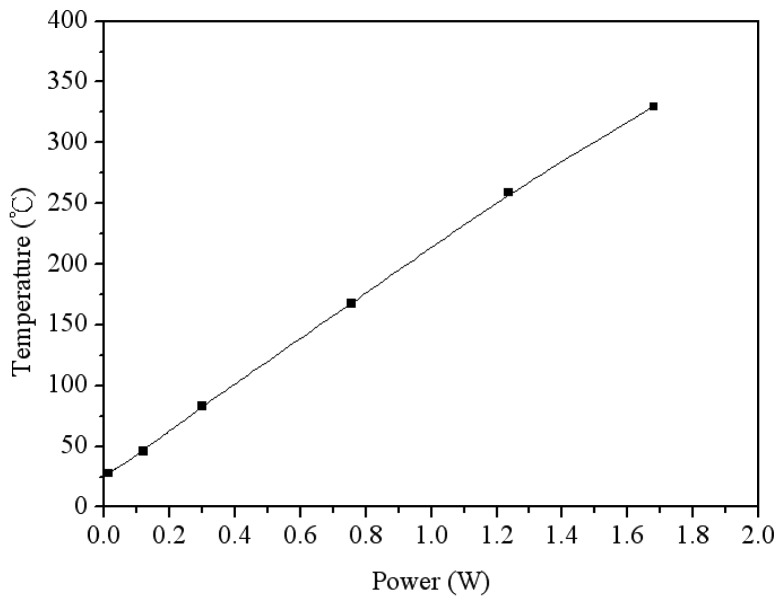
Measured results of heating temperature for the heater.

**Figure 11.**
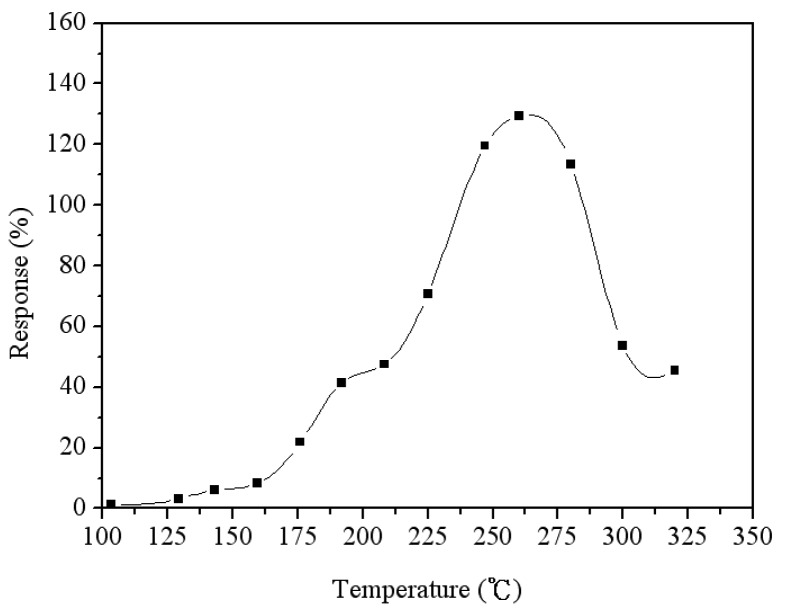
Response of the sensor at different temperatures.

The function of the heater in the sensor was tested using the infrared thermometer. Figure 10 shows the tested results of heating temperature for the heater. When supplying a power of 1.24 W, the heater generated a heating temperature of 265 °C. To characterize the optimum working temperature of the acetone microsensor, the sensor was tested under different temperatures. The heater provided different temperatures to the sensor, and the test chamber maintained at a constant 3 ppm acetone. The capacitance of the sensor was recorded using the LCR meter Figure 11 shows the response of the sensor at different temperatures. The response is defined as, $\frac{c-c_{0}}{c_{0}}\times 100\%$, where *C* is the capacitance variation of the sensor reacted with acetone gas and *C_0_* is the original capacitance of the sensor. The results showed that the optimum working temperature of the acetone microsensor was 265 °C.

**Figure 12.**
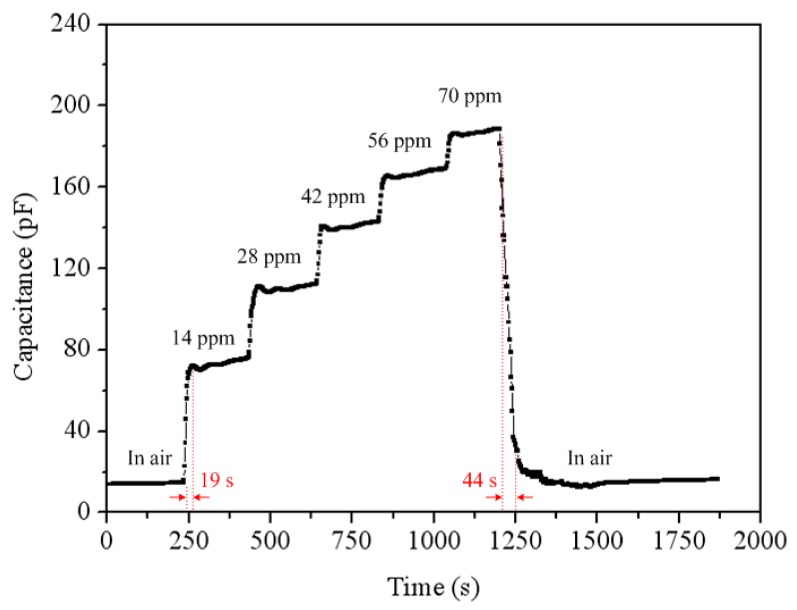
Reaction of the acetone sensor at 0–70 ppm acetone.

**Figure 13.**
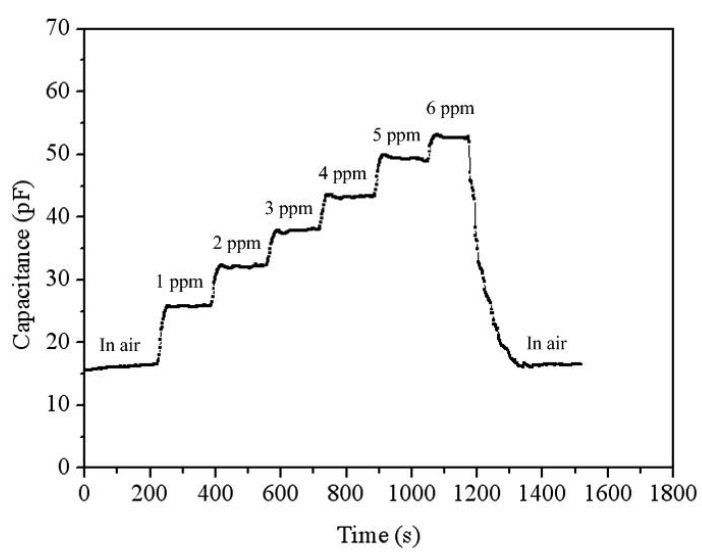
Reaction of the acetone sensor at 0–6 ppm acetone.

**Figure 14.**
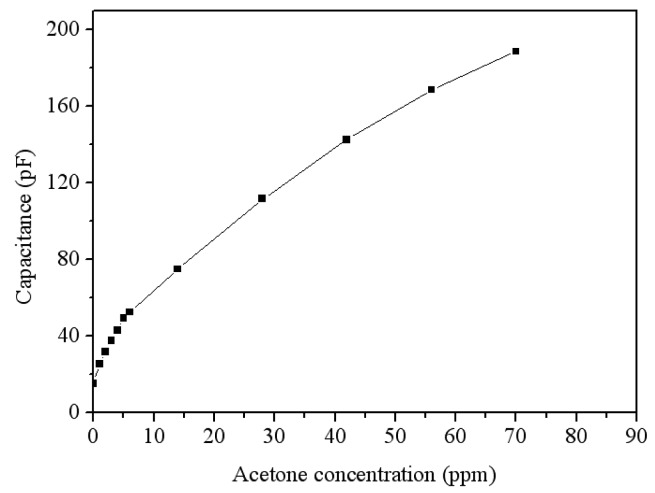
Relation between capacitance and acetone concentration for the acetone sensor.

To characterize the capacitance variation of the acetone sensor, the sensor without the oscillator circuit was tested. The acetone sensor was set in the test chamber, and the heater supplied a working temperature of 265 °C to the sensor. The LCR meter recorded the capacitance variation of the sensor under different acetone concentration. Figure 12 presents the reaction of the acetone sensor at 0–70 ppm acetone. The nonlinear envelope of the peaks in Figure 12 was due to the time intervals adopted. The results showed that the initial capacitance of the sensor was 15.5 pF, and its capacitance changed to 188.6 pF at 70 ppm acetone. As shown in Figure 12, the sensor had a response time of 19 s at 14 ppm acetone and a recovery time of 44 s at 70 mm acetone. Figure 13 presents the reaction of the acetone sensor at 0-6 ppm acetone. The results depicted that the acetone sensor had a capacitance of 26 pF at 1 ppm acetone and a capacitance of 32 pF at 2 ppm acetone. Figure 14 shows the relation between the capacitance and acetone concentration for the acetone sensor, and the results are obtained in accordance with the data in Figures 12 and 13. The capacitance of the acetone sensor increased from 26 to 188.6 pF as the acetone concentration varied from 1 to 70 ppm. The curve of capacitance in Figure 14 reveals a nonlinear variation that results from the intrinsic characteristics of α-Fe_2_O_3_ material.

**Figure 15.**
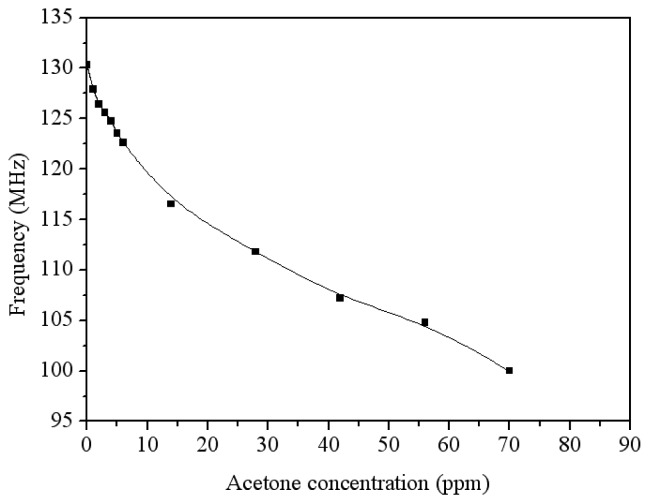
Output frequency of the acetone sensor with the oscillator circuit.

The acetone sensor with the oscillator circuit was set in the test chamber and measured the output signal. The oscillator circuit converted the capacitance of the acetone sensor into the output frequency. The power supply provided a bias voltage of 3 V to the oscillator circuit, and the heater supplied a working temperature of 265 °C to the sensor. The spectrum analyzer measured the output frequency of the oscillator circuit. Figure 15 shows the measured results of output frequency for the acetone sensor with the circuit. The results revealed that the output frequency of the acetone sensor was 130.3 MHz in air, and the sensor had an output frequency of 128 MHz at 1 ppm acetone and an output frequency of 100 MHz at 70 ppm acetone. The curve of output frequency in Figure 15 reveals a nonlinear variation that results from the oscillator circuit. The phenomenon could be improved by modifying the design of oscillator circuit.

**Figure 16.**
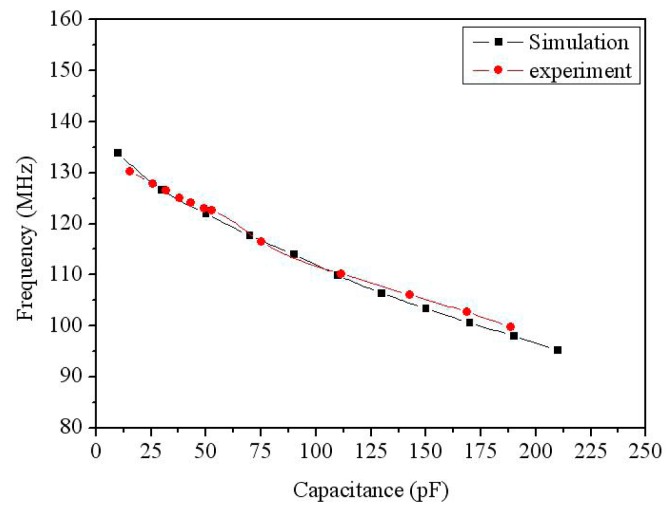
Relation between output frequency and capacitance for the acetone sensor.

To compare the simulation and experiment of output frequency for the acetone sensor, the relation between the output frequency and capacitance was plotted as shown in Figure 16, where the data of experiment was combined the results in Figure 14 with Figure 15, and the data of simulation was from the results in Figure 3. As shown in Figure 16, the experimental results are in agreement with the simulated results. The output frequency of the sensor was more linearity in the capacitance rang of 75–185 pF.

**Figure 17.**
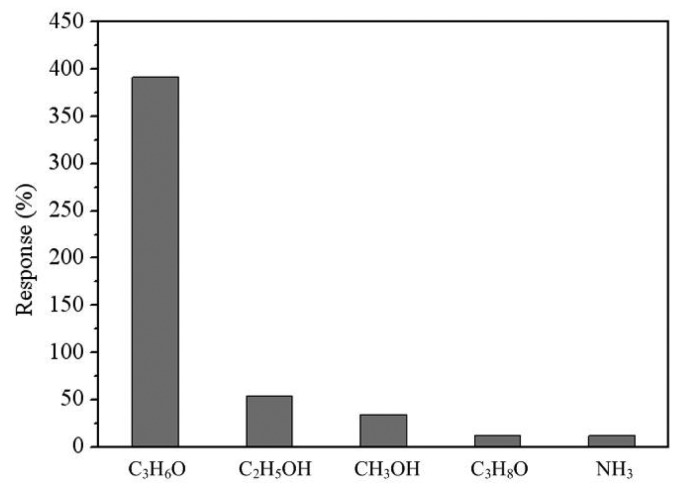
Response of the sensor under different gases.

To understand the selectivity of the sensor, it was tested with different gases. Figure 17 shows the response of the sensor under acetone, ethanol, methanol, isopropyl alcohol and ammonia. In this measurement, the concentration of all gases was controlled at 15 ppm and the working temperature of the sensor was 265 °C. The results showed that the sensor for acetone had a best response of 385%. Therefore, the sensor had an excellent selectivity for sensing acetone.

## 6. Conclusions

An acetone microsensor with a polysilicon heater and a ring oscillator circuit has been fabricated using the commercial 0.18 μm CMOS process and a post-processing step. The sensitive material was α-Fe_2_O_3_ prepared by a hydrothermal method. A heater was used to provide a optimum working temperature of 265 °C for the acetone sensor. When the α-Fe_2_O_3_ sensitive material adsorbed or desorbed acetone gas, the sensor generated a change in capacitance. The ring oscillator circuit converted the capacitance variation of the sensor into the output frequency. The post-process employed a wet etching to remove the sacrificial oxide layer and coated the sensitive material on the interdigitated electrodes. The experimental results showed that the capacitance of the acetone sensor changed from 15.53 to 188.58 pF as the acetone concentration increased from 0 to 70 ppm. The acetone sensor with the ring oscillator circuit had an output frequency of 128 MHz at 1 ppm acetone and an output frequency of 100 MHz at 70 ppm acetone.
